# PYK2 promotes HER2-positive breast cancer invasion

**DOI:** 10.1186/s13046-019-1221-0

**Published:** 2019-05-22

**Authors:** Shaymaa IK. Al-Juboori, Jayakumar Vadakekolathu, Sarra Idri, Sarah Wagner, Dimitrios Zafeiris, Joshua RD. Pearson, Rukaia Almshayakhchi, Michele Caraglia, Vincenzo Desiderio, Amanda K. Miles, David J. Boocock, Graham R. Ball, Tarik Regad

**Affiliations:** 1 The John van Geest Cancer Research Centre, School of Science and Technology, Nottingham Trent University, Clifton Lane, Nottingham, NG11 8NS UK; 2 Department of Experimental Medicine, University of Campania “Luigi Vanvitelli”, 80138 Naples, Italy; 3 Department of Precision Medicine, University of Campania “Luigi Vanvitelli”, 80138 Naples, Italy; 4 Department of Biology, College of science for women, University of Baghdad, Baghdad, Iraq

**Keywords:** Pure HER2 breast Cancer, Metformin, PYK2/PTK2B, Chemoresistance, Breast cancer stem cells

## Abstract

**Background:**

Metformin, a biguanide, is one of the most commonly prescribed treatments for type 2 diabetes and has recently been recommended as a potential drug candidate for advanced cancer therapy. Although Metformin has antiproliferative and proapoptotic effects on breast cancer, the heterogenous nature of this disease affects the response to metformin leading to the activation of pro-invasive signalling pathways that are mediated by the focal adhesion kinase PYK2 in pure HER2 phenotype breast cancer.

**Methods:**

The effect of metformin on different breast cancer cell lines, representing the molecular heterogenicity of the disease was investigated using in vitro proliferation and apoptosis assays. The activation of PYK2 by metformin in pure HER2 phenotype (HER2+/ER−/PR-) cell lines was investigated by microarrays, quantitative real time PCR and immunoblotting. Cell migration and invasion PYK2-mediated and in response to metformin were determined by wound healing and invasion assays using HER2+/ER−/PR- *PYK2* knockdown cell lines. Proteomic analyses were used to determine the role of PYK2 in HER2+/ER−/PR- proliferative, migratory and invasive cellular pathways and in response to metformin. The association between PYK2 expression and HER2+/ER−/PR- patients’ cancer-specific survival was investigated using bioinformatic analysis of *PYK2* expression from patient gene expression profiles generated by the Molecular Taxonomy of Breast Cancer International Consortium (METABRIC) study. The effect of PYK2 and metformin on tumour initiation and invasion of HER2+/ER−/PR- breast cancer stem-like cells was performed using the in vitro stem cell proliferation and invasion assays.

**Results:**

Our study showed for the first time that pure HER2 breast cancer cells are more resistant to metformin treatment when compared with the other breast cancer phenotypes. This drug resistance was associated with the activation of PTK2B/PYK2, a well-known mediator of signalling pathways involved in cell proliferation, migration and invasion. The role of PYK2 in promoting invasion of metformin resistant HER2 breast cancer cells was confirmed through investigating the effect of *PYK2* knockdown and metformin on cell invasion and by proteomic analysis of associated cellular pathways. We also reveal a correlation between high level of expression of *PYK2* and reduced survival in pure HER2 breast cancer patients. Moreover, we also report a role of PYK2 in tumour initiation and invasion-mediated by pure HER2 breast cancer stem-like cells. This was further confirmed by demonstrating a correlation between reduced survival in pure HER2 breast cancer patients and expression of *PYK2* and the stem cell marker *CD44*.

**Conclusions:**

We provide evidence of a PYK2-driven pro-invasive potential of metformin in pure HER2 cancer therapy and propose that metformin-based therapy should consider the molecular heterogeneity of breast cancer to prevent complications associated with cancer chemoresistance, invasion and recurrence in treated patients.

**Electronic supplementary material:**

The online version of this article (10.1186/s13046-019-1221-0) contains supplementary material, which is available to authorized users.

## Backgound

Cancer cells are constantly developing cellular mechanisms that confer resistance to chemotherapeutic compounds, and this leads to cancer recurrence and decreased survival in cancer patients [1, 2]. This therapeutic limitation is also observed in advanced breast cancer, where cancer cells escape the cytotoxic effects of chemotherapies by developing multiple drug resistance [3]. Metformin, a drug that is used for the treatment of type 2 diabetes, has been proposed as an alternative therapy of advanced breast cancer [4–7]. The interest in metformin as therapeutic compound, is based on meta-analyses that indicated that patients with type 2 diabetes have a reduced incidence of pancreatic, colorectal and breast cancers. An improvement in survival was also reported in metformin-treated type 2 Diabetes patients with colorectal, lung and liver cancers [8–11]. Although, in vitro studies of metformin effect on breast cancer cell lines confirmed the anti-proliferative potential of this drug [6, 12, 13], it is not yet clear whether the molecular heterogeneity of this disease interferes with the response to metformin treatment [14, 15].

The protein tyrosine kinase PYK2, also known as PTK2B, is a non-receptor tyrosine kinase involved in the regulation of cell growth, proliferation, survival, migration and invasion [16–18]. As a member of FAK (Focal Adhesion Kinase) family of kinases, PYK2 functions as a linker between transmembrane glycoproteins and actin cytoskeleton [19]. In cancer, PYK2 plays an important role in tumourigenesis, invasion and metastasis and its high level of expression in patients’ tumours correlates with poor outcomes [20–27]. In breast cancer, PYK2 expression is increased in early and advanced ductal breast cancer and correlates with an increased expression of HER2 [28]. Although this involvement could be explained by the role of PYK2 in breast cancer migration and invasion [24], its role in breast cancer chemoresistance and potentially in associated breast cancer recurrence is unknown.

In this study, we demonstrate that breast cancer cells characterised by the HER2 phenotype (HER2+/ER−/PR-) are more resistant to metformin treatment. We demonstrate that unlike cells that are luminal A, luminal B, claudin low or basal-like, HER2+/ER−/PR- (HER2) cells exhibit increased cell proliferation and decreased apoptosis, in response to metformin. More importantly, we report that metformin treatment leads to an increase in PYK2 expression that is associated with cell invasion of HER2+/ER−/PR- cells*.* These results were confirmed by proteomic analysis which indicated that several pathways involved in cancer invasion were affected following *PYK2* knockdown. Furthermore, analysis of *PYK2* expression from HER2+/ER−/PR- breast cancer patients indicates a correlation between high expression levels of *PYK2* and patients’ reduced survival. Finally, we show a role of PYK2 in cancer initiation and in regulating self-renewal and invasion of HER2+/ER−/PR- cancer stem-like cells and in response to metformin. Overall, this study suggests that future applications of metformin in breast cancer therapy should consider the molecular heterogeneity of this disease, and particularly the HER2 breast cancer phenotype, to prevent the development of a more aggressive form of breast cancer, associated with metformin-based therapy.

## Methods

### Cell lines, growth conditions and metformin treatment

The human breast cancer cell lines BT-474, MCF-7, MDA-MB-231 and MDA-MB-468 and SkBr-3 were purchased from ATCC (ATCC-HTB-20, ATCC-HTB-22, ATCC-HTB-26, ATCC-HTB-132 and ATCC-HTB-30). The breast cancer cell line MDA-MB-453 was purchased from Deutsche Sammlung von Mikroorganismen und Zellkulturen (DSMZ) (ACC65). All cell lines were cultured in their dedicated media. The cell lines were used, for the experiments, at a very low passage and were regularly morphologically checked. BT-474 cell line was cultured in Hybri-Care media. Minimum Essential Medium Eagles (EMEM) from SLS (Lonza) was used to culture the MCF-7 cell line with addition of 0.01 mg/ml insulin solution (SIGMA). LEIBOVITZ (L-15) media complemented with 1% L-Glutamine (SLS (Lonza)) was used for both MDA-MB-231, MDA-MB-468 and MDA-MB-453. While, Mc Coy’s 5A was used for culturing the SkBr-3 cell line. 10% fetal bovine serum (FBS) was added to all types of media as supplementary agent in accordance with ATCC recommendations. BT-474, MCF-7, and SKBR-3 were incubated at 37 °C with 5% CO_2_, while MDA-MB-231 and MDA-MB-468 are incubated at 37 °C, in humidified atmosphere without CO_2_. Metformin (1, 1-Dimethylbiguanide hydrochloride 97%, D150959-5G, Sigma-Aldrich, UK) was dissolved in culture media at concentrations of 1 M and 10^-2^M was used as a stock to prepare different concentrations for treatment of cells (1, 2, 5, 10, 15, 20, 25) mM and (0.01, 0.05, 0.1, 0.5) mM consecutively.

### Generation of PYK2 knockdown cell lines

Lentiviral PTK2B shRNA plasmids were purchased from Sigma-Aldrich (MISSION shRNA Plasmid DNA protein tyrosine kinase 2 beta (PTK2B/PYK2) SHCLND-NM_004103. 3-763S1C1 & NM_004103. 3-4018S21C1). The lentiviral packaging mix was also purchased from Sigma (SHP001). Plasmids were transfected using Lipofectamine™ 3000 Reagent (L3000001, ThermoFisher Scientific) and following manufacturer recommendations. The lentiviral particles used to infect SkBr3 and MDA-MB-453 were produced corresponding to the manufacturer recommendations and as previously described [29].

### Cell proliferation assay

**Fig. 1:**
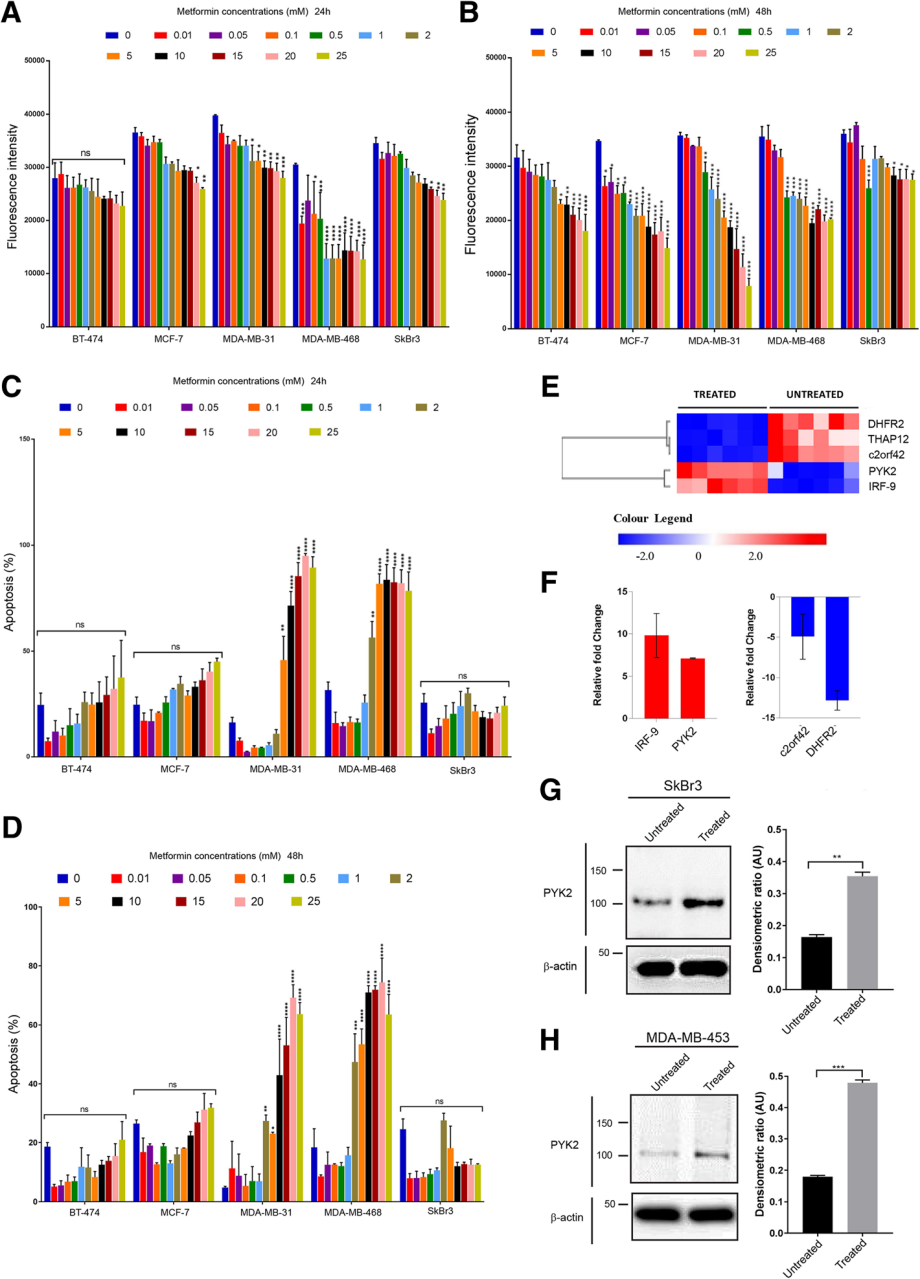
Effect of metformin on cell proliferation and apoptosis of breast cancer cell lines representing different phenotypes of breast cancer. **a**, **b** Effect of different concentrations of metformin on cell proliferation of BT-474, MCF-7, MDA-MB-231, MDA-MB-468 and SkBr3, 24 h and 48 h post-treatment. *N* = 3 (6 replicates). **c**, **d** Effect of different concentrations of metformin on apoptosis of BT-474, MCF-7, MDA-MB-231, MDA-MB-468 and SkBr3, 24 h and 48 h post-treatment. N = 3 (2 replicates). The statistical values are provided as Additional file 4: Data S1. **e** Heatmap of microarray analysis showing upregulated (in red) and downregulated genes (in blue) in untreated vs. treated cells. Bonferroni corrected *P* value ≤0.05. *N* = 6 (6 replicates). **f** RT-PCR analysis of relative gene expression of selected IRF-9 and PYK2 (Upregulated), and c2orf42 and DHFR2 (downregulated). N = 3 (3 replicates). **g**, **h** Immunoblot images representing PYK2 expression in metformin treated and untreated SkBr3 and MDA-MB-453 cell lines. Densitometric ratio is measured by Arbitrary Units (AU). Student t-test, **P = 0.0030 and ***P = 0.0006. N = 3 (3 replicates)

Cell proliferation described in Fig. 1 was measured using The CyQUANT® NF assay (Molecular Probes™ C35007) and following manufacturer recommendations. Fluorescence intensity was measured using a fluorescence microplate reader TECAN ULTRA fluorescence spectrophotometer with excitation at ~ 485 nm and emission detection at ~ 530 nm (Infinite® 200 PRO). Cell proliferation described in Fig. 3 was performed using the xCELLigence system, and cell index was measured following manufacturer recommendations. The results were analysed using RTCA software (Real-time cell analysis software Xcelligence).

### Cell apoptosis assay

Cells were plated in 6-well plates at a density of 1 × 10^5^ cells/well in 2 ml of media and incubated for 24 h. The media was removed, and the cells were treated with different concentrations of metformin (0-25 mM) and incubated for 24 h and 48 h, respectively. The cells were collected after 24 and 48 h of treatment, and the number of cells were counted by the Chemometec Nucleo Counter® NC-250 TM (Chemometec). The tubes were incubated in the dark for 15 min and 350 μL of Annaxin V Binding Buffer and 10 μL of 1/20 of Propidium Iodide solution (PI) were added. The proportion of living cells, early apoptotic cells, and necrotic cells were determined using a flow cytometer (Beckmen Coulter Gallios Flow Cytometer).

### Immunoblotting

Immunoblotting was performed as previously described [30]. In brief, metformin-treated and untreated breast cancer cells were collected, washed with 1X PBS, lysed in 1X solution containing 50 mM Tris-HCl (pH 6.8), 100 mM dithiothreitol, 2% (w/v) SDS, 0.1% (w/v) bromophenol blue and 10% (v/v) glycerol, and loaded on Tris/glycine SDS-polyacrylamide gels. The proteins on gels were transferred onto Amersham Hybond-P PVDF membranes (GE Healthcare, Life science, Chalfont, UK). Membranes were blocked with 10% (w/v) Marvel milk/tris-buffered saline (TBS) solution with 0.01% (v/v) Tween-20 (TBST), washes in TBST and incubated with primary antibodies (PYK2 Antibody (9H12L1), 1: 500 for IB, 700183, Invitrogen, Thermo Fisher Scientific; anti-b-actin, 1:5000 for IB, A5441, Sigma-Aldrich, St Louis, MO, USA) (in blocking solution) overnight at 4 °C, and followed by washing and incubation with secondary antibodies for 1 h at room temperature, prior to visualisation using Rapid Step ECL reagent (Calbiochem, Billerica, MA, USA) and a CCD camera -Western blot imager (Syngene).

### Gene expression and microarrays analyses

Microarrays analysis were performed from RNAs extracted from cells using STAT 60 and RNeasy Mini Kit (50) from QIAGEN and following the manufacturer’s instructions protocol. 200 ng of total RNA was labelled using Agilent low input QuickAmp one-color labelling kit. Quality of labelling checked using nanodrop 8000 and hybridised onto an Agilent Human GE 4x44K v2 microarrays at 65 °C for 16 h. Slides were washed and scanned using a GenePix pro- 4100A scanner, quality controls and raw data of the scanned images were generated using Agilent feature extraction software V11.0. Raw data was normalised using Partek Genomic Suit V 6.0 and used for ANN analysis. Microarrays gene expression data were used for pathways analysis using Panther Pathway. Gene expression of *IRF-9, PTK2B, C2ORF42* and *DHFR2* were assessed by Real-time quantitative PCR using the following primers: FH1_IRF9:5′-CTCAGAAAGTACCATCAAAGC-3′; RH1_IRF9:5′- TCATTATTGAGGGAGTCCTG-3′; FH1_PYK2: 5′-AATGCACTTGACAAGAAGTC-3′; RH1_PYK2:5′-GCTTTAAGTTCTCCTGCATC-3′; FH1_C2orf42: 5′-AGCTTTTGTTCGGAAAGATG-3′; RH1_C2orf42: 5′-GCATCTCTGGGGTATCTAAG-3′; FH1_DHFR2:5′-CGCTGTGTCCCAAAACATGG-3′; RH1_DHFR2: 5′- GAATTCATTCCTGAGCGGCG-3′. The gene expression microarrays data was deposited in the online database ArrayExpress under the accession number: E-MTAB-7737.

### Mass spectrometry analysis

Cell lysates (100 μg) were reduced/alkylated and digested as described previously. Next, samples were de-salted and concentrated using HyperSep C_18_ spin tips (10-200 μL size) (Thermo Scientific) using the manufacturer recommended protocol. Samples were dried and resuspended in 5% acetonitrile + 0.1% formic acid for MS analysis in both SWATH and IDA (information dependent acquisition) modes.

### Mass spectrometry

Each sample was analysed on a SCIEX TripleTof 6600 mass spectrometer coupled in line with an Eksigent ekspert nano LC 425 system running in microflow as described previously [31] with minor modifications. In brief, 6 μg (3 uL) of sample was injected via trap/elute. The following linear gradients were used (5 uL/min): mobile phase B (acetonitrile + 0.1% formic acid) over mobile phase A (0.1% formic acid) as follows: SWATH (57 min run) increasing from 3 to 30% over 38 min, 30 to 40% over 5 min, 40 to 80% over 2 min; IDA (87 min run) increasing from 3 to 30% over 68 min, 30 to 40% over 5 min, 40 to 80% over 2 min followed by wash and re-equilibration.

### Library generation, spectral alignment and fold change analysis

IDA mass spectrometry files were searched using ProteinPilot software 5.0.1 (SCIEX) with the following search criteria: exclude biological modifications, thorough ID, searching the UniProt Swiss-Prot human database (March 2018 release). The resulting ion library file was aligned using endogenous peptides to the SWATH files and processed using the OneOmics cloud processing platform (SCIEX, Warrington UK) as described previously [32].

### Sphere formation assay

Metformin-treated and untreated SKBR-3 cells were harvested and counted and then cultured in complete Mammocult™ medium (Stem Cell Technologies) in ultra-low attachment plates (Corning) at a density of 2 × 10^4^ viable cells/mL. The number of spheres that developed in each condition was counted after 10 days culture in a 5% CO_2_, humidified incubator at 37 °C.

### Flow cytometry

#### Analysis of CD44^+^/CD24^−/low^ breast CSC surface markers expression

The spheres were collected by gentle centrifugation and dissociated using trypsin-EDTA. The resulting single cells were then washed in PBS prior to the addition of fluorochrome-conjugated monoclonal antibodies against human CD44 (APC; clone IM7) and CD24 (PE; clone ML5) (Biolegend) for 30 min at 4 °C in the dark. The LIVE/DEAD™ Fixable Violet Dead stain (Invitrogen) was used for dead cells exclusion. The labelled cells were then washed in PBS, re-suspended in Coulter Isoton™ diluent and analysed on a Gallios™ flow cytometer using Kaluza™ v1.3 acquisition and analysis software (Beckman Coulter).

#### AldeRed ALDH detection assay

The AldeRed™ Detection Assay (SCR150, Merck Millipore) was used in accordance with the manufacturer’s instructions. Briefly, 2 × 10^5^ cells were incubated with the AldeRed 588-A substrate for 40 min at 37 °C, baseline fluorescence was established using negative control samples incubated with the ALDH1 inhibitor, diethylaminobenzaldehyde (DEAB). Subsequently, cells were centrifuged, re-suspended in AldeRed assay buffer and analysed on a Gallios™ flow cytometer using Kaluza™ v1.3 acquisition and analysis software (Beckman Coulter).

#### Cell migration and invasion assays

A wound-healing assay (Scratch assay) was applied to different breast cancer PYK2 knockdown cell lines following this protocol. Briefly, the cells were counted to 1 × 10^6^ cells and seeded in 6 well plates with 2 mL of cell dedicated media supplemented with Puromycin (3 μg/mL) as non-treated cells and supplemented with Puromycin and Metformin (1 μL/mL) as treated cells. The following day the media was removed and instead serum-free media was added (to prevent cell proliferation). After 24 h, the media was removed, and the cells were washed with 1 mL of DPBS for each well. Scratches were performed vertically by using a 200 μL pipette tip. The cells were washed twice with 0.5 mL (DPBS) and fresh serum-free media was added to each well (with or without metformin). The measurements were carried out at time 0 and 48 h by utilising the LCM and Axiovision software for imaging the scratches. The invasion assay has been implemented according to Cultrex® BME Cell Invasion Assay 96 well kit (R&D Systems) manufacturer protocol. This assay has been performed using untreated and metformin-treated MDA-MB-453 and SkBr3 control and PYK2 knockdown cell lines following the manufacturer’s recommendations. For the stem cell invasion assay, untreated and treated spheres were dissociated to obtain single cells for the invasion assay. The cells in the bottom chamber were labelled by Calcein AM and the quantification of the number of cells was performed using the plate reader Infinite M200 Pro TECAN at 585 nm excitation and 520 nm emission.

### Statistical analysis

The analysis was performed using patient gene expression profiles generated by the Molecular Taxonomy of Breast Cancer International Consortium (METABRIC) study [33] using Illumina HT-12 v3.0 Gene Expression BeadChip. For the here conducted study, patients were selected with a recorded overall survival (OS) (censored or complete) of ≤5 years. This resulted in a total number of 610 patients. Of these 610 patients, 84 were a pure HER2+ population. Kaplan-Meier analysis was performed on a median separation of the population by gene expression, resulting in equal patient numbers per group. Median survival time is shown in years for each group. Correlation analysis was performed using Pearson correlation. A *p*-value below 0.05 was considered significant. The probe ID used for the PTK2B (PYK2) analysis was ILMN_1732318.

## Results

### HER2+/ER−/PR- breast cancer cells are resistant to metformin treatment

To investigate the effect of metformin on the different subtypes of breast cancer, we investigated its effect on the proliferation and apoptosis of BT-474 (Luminal B), MCF-7(Luminal A), MDA-MB-231(Claudin low), MDA-MB-468 (Basal-like) and SkBr3 (HER2) cell lines. The cells were treated with increasing concentrations of metformin: 0.01, 0.05, 0.1, 0.5, 1, 2, 5, 10, 15, 20 and 25 mM, and a proliferation assay was performed 24 h and 48 h following treatment (Fig. 1 a and b). All metformin-treated breast cancer cell lines showed a decreased proliferation 24 h and 48 h post-treatment, however, we observed that the proliferation of the HER2+/ER−/PR- breast cancer cell line was the least affected by metformin 48 h post-treatment (Fig. 1b). Apoptosis experiments that were performed and analysed by flow cytometry using the apoptosis marker annexin V, indicated that SkBr3 cells and although not biologically significant, had less apoptotic cells 24 h and 48 h following treatment, and when compared to MCF-7, MDA-MB-231 and MDA-MB-468 breast cancer cell lines (Fig. 1c and d). Further apoptosis analysis using another HER2 breast cancer cell line MDA-MB-453 resulted in similar results to the ones obtained with SkBr3 (Additional file 1: Figure S1A) These results demonstrate that the SkBr3 breast cancer cells, characterised by the HER2+/ER−/PR- phenotype (HER2), are more resistant to metformin as they were less affected by the apoptotic effect of metformin and could maintain a higher proliferation ability compared to the other breast cancer cell lines.

### Metformin promotes the expression of PYK2 in HER2+/ER−/PR- cells

The resistance of SkBr3 cells to metformin is likely to be driven by the expression of genes and associated cellular pathways. To investigate this, we performed microarrays analysis of mRNAs from 48 h metformin-treated (1 mM concentration) and untreated SkBR3 cells. Several genes that were upregulated and downregulated in response to metformin treatment were identified (Fig. 1e). The results of microarray analysis were confirmed by qRT-PCR through investigating the levels of gene expression of *PYK2* (Protein Tyrosine Kinase 2 Beta) and *IRF9* (Interferon regulatory factor 9) that were found upregulated, and *C2ORF42* (Chromosome 2 Open Reading Frame 42) and *DHFR2* (dihydrofolate reductase 2) that were found downregulated (Fig. 1f). PYK2 role in tumourigenesis and in breast cancer invasion is well known [24, 34], and thus, we selected this molecule for further studies to determine its role in HER2+/ER−/PR- breast cancer and in response to metformin treatment. To confirm the increased expression of PYK2 in HER2+/ER−/PR- breast cancer cells following metformin treatment, immunoblotting experiments were performed with PYK2 antibody and using whole cell extracts from SkBr3 and MDA-MB-453 breast cancer cell lines. The MDA-MB-453 is another HER2+/ER−/PR- breast cancer cell that was used for this study. The results confirmed the increase of PYK2 expression at the protein level and in response to metformin treatment (Fig. 1g and h). This increase was also observed for the triple positive breast cancer cell line BT-474 but was nor observed for the other cell lines tested (Additional file 1: Figure S1 D and E).

### PYK2 promotes migration and invasion of HER2+/ER−/PR- breast cancer cells and in response to metformin treatment

**Fig. 2:**
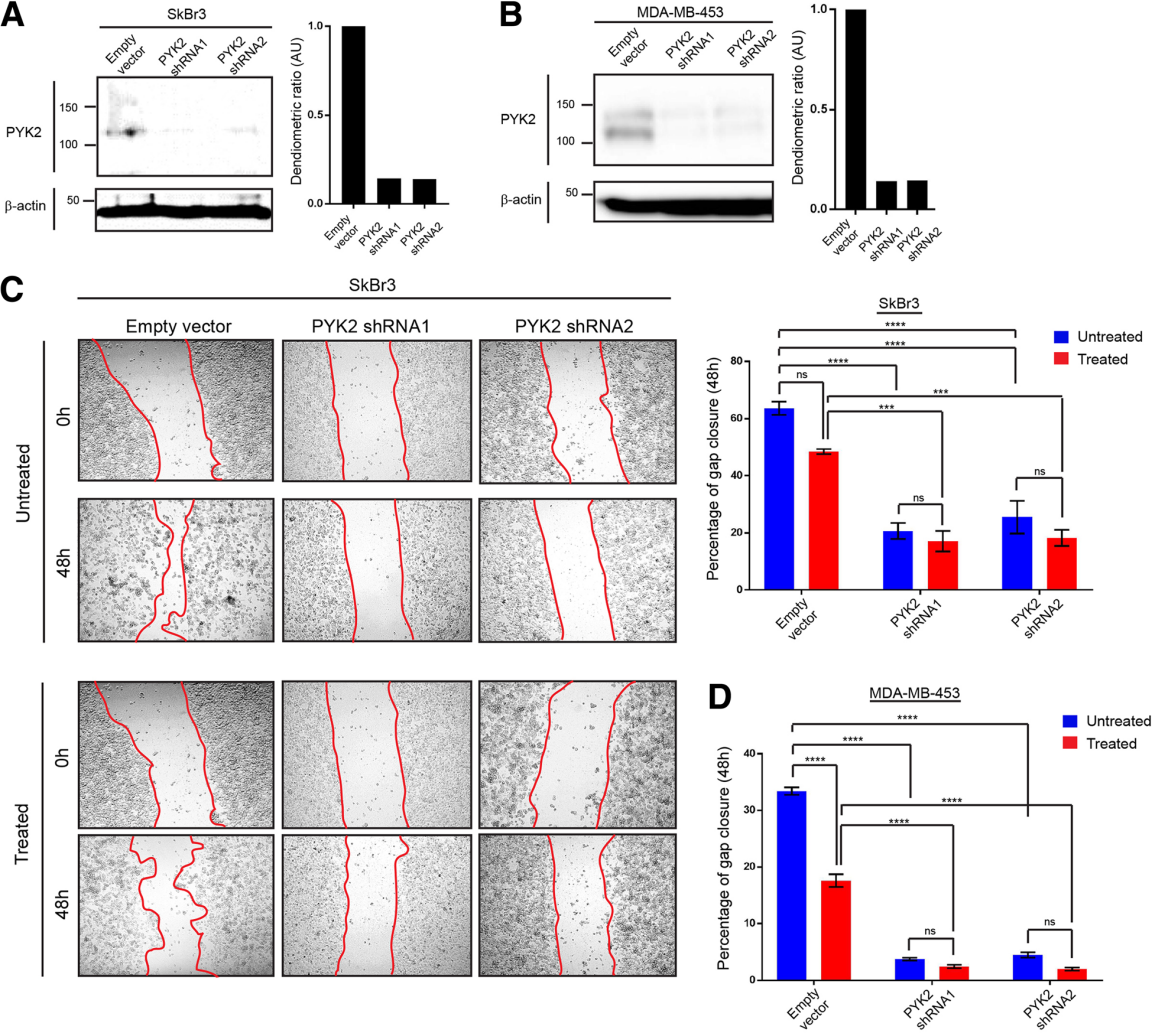
Effect of *PYK2* knockdown on cell migration of the HER2+/ER−/PR- breast cancer cell lines SkBr3 and MDA-MB-453. **a**, **b** Immunoblot images representing PYK2 expression SkBr3 and MDA-MB-453 control (pLKO.1 empty vector) and *PYK2* knockdown cells. N = 3 (3 replicates). **c** Wound healing assay (scratch assay) using SkBr3 control and *PYK2* knockdown metformin treated and untreated cells, and the corresponding data quantifying gap closure at time points 0 and 48 h following scratching. Anova ****P = < 0.0001, ***P = 0.0003 (Empty vector vs. PYK2 shRNA1), ***P = 0.0004 (Empty vector vs. PYK2 shRNA2). **d** Data quantifying gap closure at time points 0 and 48 h following scratching Wound healing assay (scratch assay) using MDA-MB-453 control and PYK2 knockdown metformin treated and untreated cells. Anova ****P = < 0.0001. *N* = 3 (2 replicates)

**Fig. 3:**
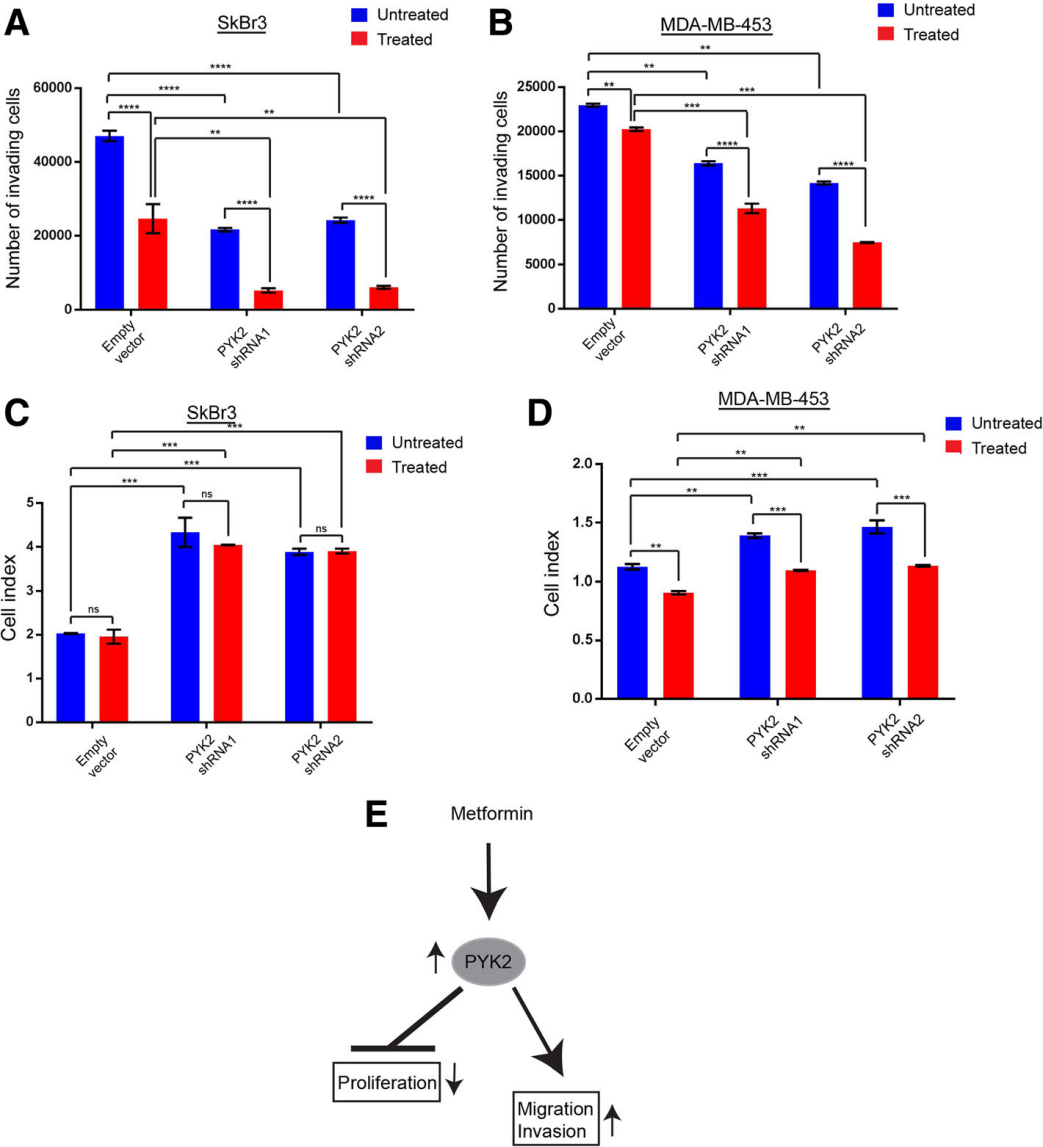
Effect of PYK2 knockdown on cell proliferation and invasion of the HER2+/ER−/PR- breast cancer cell lines SkBr3 and MDA-MB-453. **a** Cell invasion assay using SkBr3 cells metformin treated and untreated and the corresponding data quantifying of number of invading cells (48 h with or without treatment). Anova ****P = < 0.0001, **P = 0.0025 (Treated empty vector vs. treated PYK2 shRNA1), ***P = 0.0032 (Treated empty vector vs. treated PYK2 shRNA2). *N* = 3 (2 replicates). **b** Cell invasion assay using MDA-MB-453 metformin treated and untreated cells, and the corresponding data quantifying number of invading cells (48 h with or without treatment). Anova ****P = < 0.0001, **P = 0.0032 (Empty vector vs. treated empty vector), **P = 0.0030 (Empty vector vs. PYK2 shRNA1), **P = 0.0017 (Empty vector vs. PYK2 shRNA1), ***P = 0.0005 (Treated empty vector vs. treated PYK2 shRNA2), ***P = 0.0005 (Treated empty vector vs. treated PYK2 shRNA1). *N* = 3 (2 replicates). **c** Cell proliferation assay using SkBr3 metformin treated and untreated cells, and the corresponding data quantified as cell index (48 h with or without treatment). Anova, ***P = 0.0001 (Empty vector vs. PYK2 shRNA1), ***P = 0.0004 (Empty vector vs. PYK2 shRNA2), ***P = 0.0002 (Treated empty vector vs. treated PYK2 shRNA1), **P = 0.0003 (Treated empty vector vs. treated PYK2 shRNA2). *N* = 3 (2 replicates). **d** Cell proliferation assay using MDA-MB-453 metformin treated and untreated cells, and the corresponding data quantified as cell index (48 h with or without treatment). Anova, **P = 0.0034 (Empty vector vs. treated empty vector), **P = 0.0010 (Empty vector vs. PYK2 shRNA1), ***P = 0.0003 (Empty vector vs. PYK2 shRNA2), **P = 0.0060 (Treated empty vector vs. treated PYK2 shRNA1), **P = 0.0022 (Treated empty vector vs. treated PYK2 shRNA1), ***P = 0.0007 (Untreated PYK2 shRNA1vs. treated PYK2 shRNA1), ***P = 0.0004 (Untreated PYK2 shRNA2vs. treated PYK2 shRNA2). N = 3 (2 replicates). **e** Schematic representation of the dual role of PYK2 in proliferation, migration and invasion of HER2+/ER−/PR- breast cancer in response to metformin

To investigate the potential role of PYK2 in migration and invasion of HER2+/ER−/PR- breast cancer cells and in response to metformin (1 mM), SkBr3 and MDA-MB-453 *PYK2* knockdown cell lines were generated (Fig. 2a and b, Additional file 1: Figure S1B and C). Cell migration and invasion investigated using scratch and cell-well invasion assays, demonstrated significant decreases in these processes in metformin-untreated *PYK2* knockdown cells, and when compared to the control (pLKO.1 empty vector) (Fig. 2 c and d, and Fig. 3 a and b). A decrease in cell migration and invasion was also observed in metformin-treated *PYK2* knockdown cells, and when compared to the control (pLKO.1 empty vector) (Fig. 2 c and d, Fig. 3 a and b, and Additional file 2: Figure S2). These results indicate that metformin-induced migration and invasion in SkBr3 and MDA-MB-453 breast cancer cell lines may require PYK2. On the contrary, *PYK2* knockdown increased cell proliferation of SkBr3 and MDA-MB-453 cells, suggesting an antiproliferative function of PYK2 in HER2+/ER−/PR- breast cancer cell lines (Fig. 3c and d, Additional file 2: Figure S2). Although metformin treatment did not affect the proliferation of SkBr3 *PYK2* knockdown cells, a significant decrease was observed in MDA-MB-453 *PYK2* knockdown cells. Collectively, these results demonstrate that PYK2 plays a dual role in tumourigenesis and cancer progression via promoting invasion and preventing proliferation of HER2+/ER−/PR- breast cancer cells (Fig. 3e).

### High level of PYK2 expression correlates with decreased survival of HER2+/ER−/PR- breast cancer patients

**Fig. 4:**
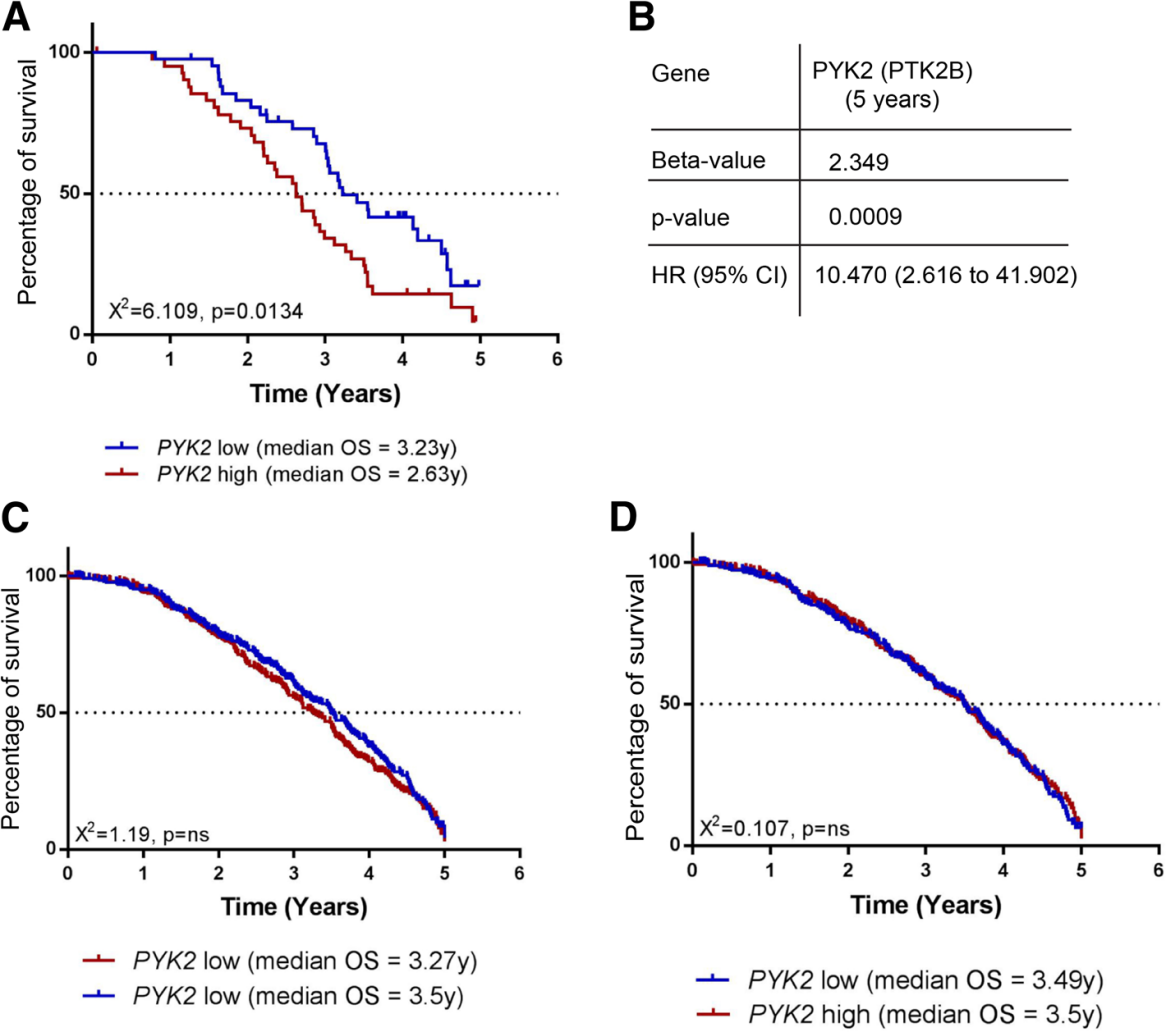
*PYK2* expression and HER2+/ER−/PR- breast cancer patients’ survival. **a**, **c**, **d** Kaplan–Meier plots representing correlations between high or low levels of *PYK2* expression and percentage of survival of HER2+/ER−/PR- breast cancer patients (**a**) Total population of breast cancer patients **c** and total populations of breast cancer patients excluding HER2+/ER−/PR- breast cancer patients **d**. **b** Cox regression analysis of correlation between *PYK2* expression and survival of HER2+/ER−/PR- breast cancer patients

Although a previous study has shown a correlation between high level of expression of PYK2 and breast cancer progression [34], the role of PYK2 in this process and specifically in HER2+/ER−/PR- breast cancer is still unclear. To examine this, the association of *PYK2* expression with cancer-specific survival in a cohort of patients with HER2+/ER−/PR- breast cancer was investigated by bioinformatics analysis of *PYK2* expression using patient gene expression profiles generated by the Molecular Taxonomy of Breast Cancer International Consortium (METABRIC) study [33]. In this analysis, the capacity of high or low expression of *PYK2* to predict clinical outcome was assessed. A significant reduction in cancer-specific survival (*χ*^2^ = 6.109, *P* = 0.0134) over a 5-year term was associated with high expression of PYK2, and when compared to cancer-specific survival in patients with low expression of PYK2 (Fig. 4a). This result was further confirmed using Cox regression analysis (Fig. 4b) Although analysis of the capacity of high or low expression of *PYK2* to predict cancer-specific survival in the total cohort of patients showed a non-statistically significant reduction associated with high expression of *PYK2* (*χ*^2^ = 1.19, p = ns) (Fig. 4c), the same analysis excluding HER2+/ER−/PR- breast cancer patients resulted in no difference in cancer-specific survival between low and high expression of PYK2 (*χ*^2^ = 1.107, p = ns) (Fig. 4d).

### Cellular pathways associated with PYK2 function in HER2+/ER−/PR- cells and in response to metformin treatment

**Fig. 5:**
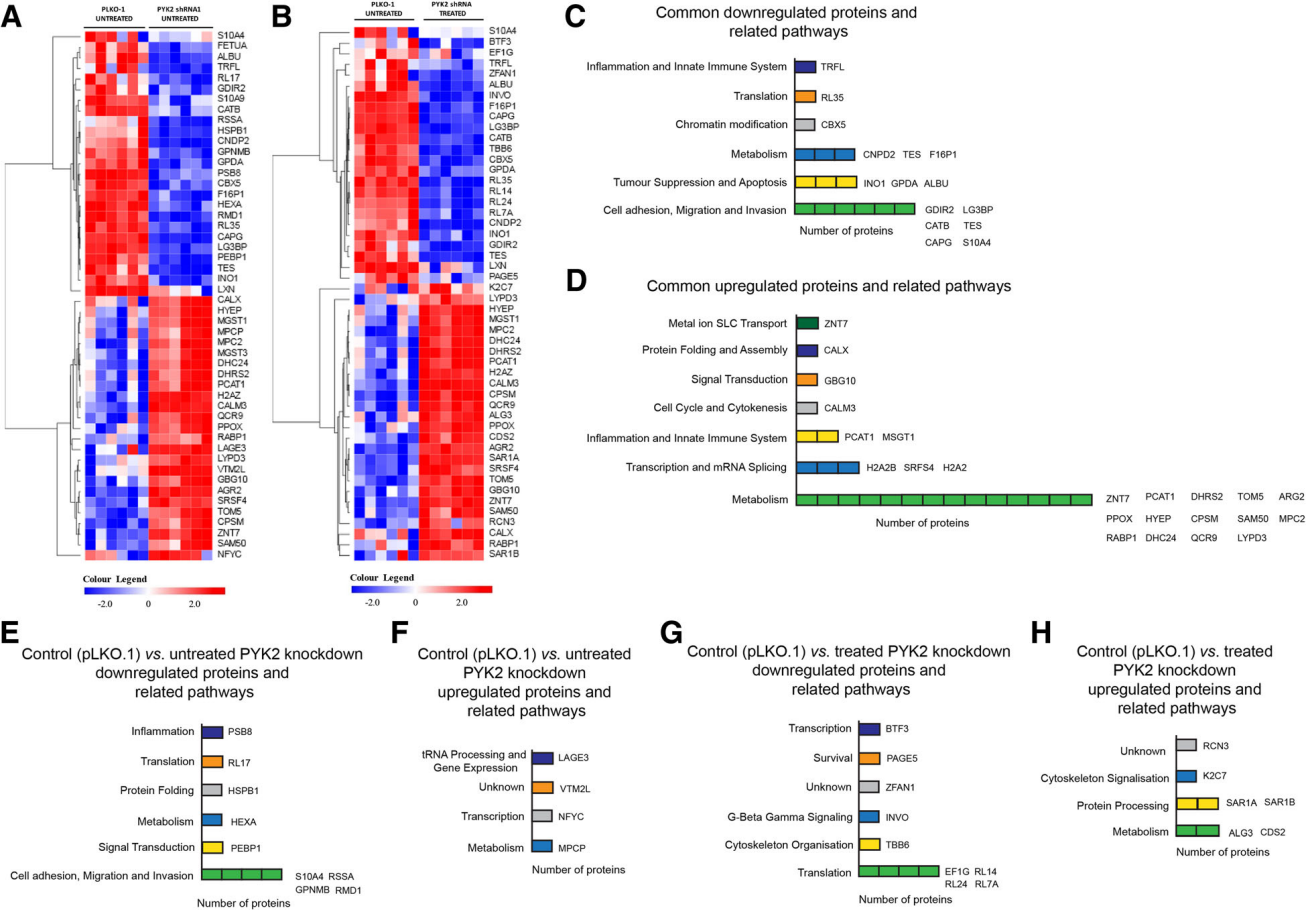
Proteomic analysis of 3546 proteins showing several upregulated and downregulated proteins and related pathways in untreated controls and metformin-treated and untreated *PYK2* knockdown SkBr3 cells. **a** Heatmap representing the top 25 upregulated and downregulated proteins in untreated control and *PYK2* knockdown SkBr3 cells. **b** Heatmap representing the top 25 upregulated and downregulated proteins in untreated control and *PYK2* knockdown metformin-treated SkBr3 cells. *N* = 1 (6 replicates) (**c**, **d**) Schematic representation of identified downregulated and upregulated proteins and related pathways that are in common and different between untreated and metformin-treated *PYK2* knockdown cells. **e**, **f** Schematic representation of identified downregulated and upregulated proteins and related pathways in untreated *PYK2* knockdown cells. **g**, **h** Schematic representation of identified downregulated and upregulated proteins and related pathways in metformin-treated *PYK2* knockdown cells

To further investigate the role of PYK2 in proliferation and invasion of HER2+/ER−/PR- breast cancer cells, we performed mass spectrometry analyses of protein extracts from untreated control (pLKO.1 empty vector) and untreated and treated *PYK2* knockdown SkBr3 cells (Fig. 4a and b). 3546 proteins were quantified by SWATH-MS and processed using OneOmics. The analyses identified several significantly differentially expressed proteins that were common to both untreated control (pLKO.1 empty vector) vs. untreated *PYK2* knockdown and untreated control (pLKO.1) vs. treated *PYK2* knockdown (Fig. 5a, b, c d and Additional file 5: Data S2). Downregulated proteins common to both groups were associated with cellular pathways such as cell adhesion, migration, invasion, tumour suppression and apoptosis (Fig. 5c), whereas, the upregulated proteins were mostly associated with cell metabolism (Fig. 5d). Proteins that were specifically found in untreated control vs. *PYK2* knockdown, and that were downregulated, are involved in cell adhesion, migration and invasion (Fig. 5e); while the upregulated ones are involved in metabolism, transcription and tRNA processing (Fig. 5f). Proteins that were specifically found in untreated control vs. treated *PYK2* knockdown and that were downregulated, are mainly involved in translation (downregulated expression) (Fig. 5g), while the upregulated ones are involved in metabolism and protein processing (upregulated expression) (Fig. 5h). Several proteins that involved in cell adhesion, migration, invasion, tumour suppression and apoptosis are also found upregulated and downregulated in MDA-MB-453 untreated control (pLKO.1) vs. untreated or treated *PYK2* knockdown (Additional file 3: Figure S3 and Additional file 6: Data S3). Although several pathways appear to be involved in PYK2 function in HER2+/ER−/PR- breast cancer cells, the above results confirm our observations of the role of PYK2 in cell migration and invasion.

### PYK2 promotes cell invasion of HER2+/ER−/PR- breast cancer stem-like cells and in response to metformin treatment

**Fig. 6:**
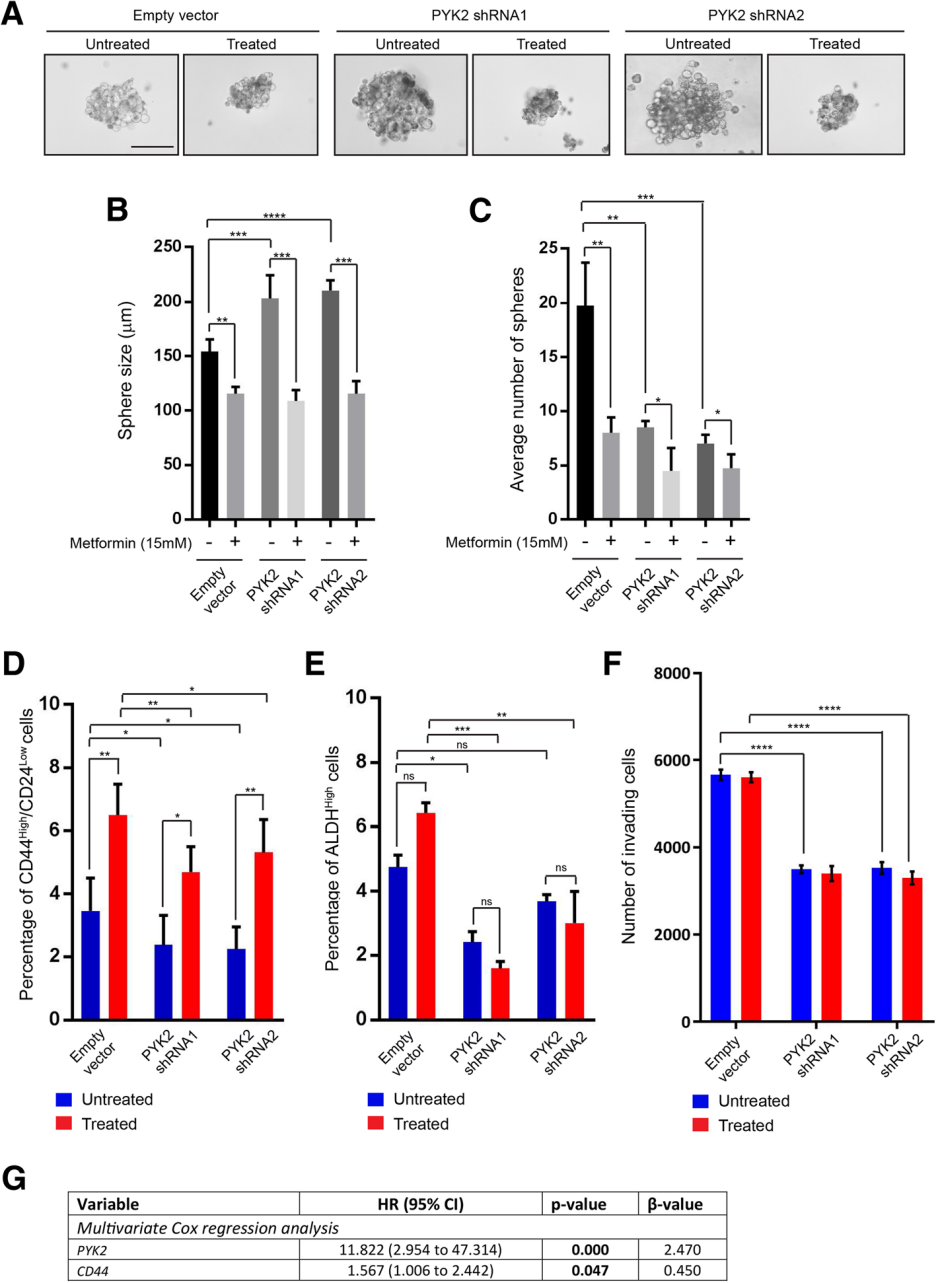
PYK2 and metformin promote the enrichment of HER2+/ER−/PR- CD44^High^/CD24^Low/−^ and ALDH^High^ breast cancer stem-like cells. **a** Images of representative spheres from 10 days sphere formation assay using SkBr3 control and PYK2 knockdowns cells metformin treated and untreated. Scale bar = 100 μm. **b** Graph representing sphere size quantification (μm) of spheres from SkBr3 control and PYK2 knockdowns cells metformin treated and untreated. Student t-test, ****P = < 0.0001, ***P = 0.0009 (Empty vector vs. PYK2 shRNA1), ***P = 0.0003 (untreated PYK2 shRNA1 vs. treated PYK2 shRNA1), ***P = 0.0002 (Treated PYK2 shRNA2vs. treated PYK2 shRNA2). N = 3 (3 replicates). **c** Graph representing average number of spheres from SkBr3 control and PYK2 knockdowns cells metformin treated and untreated. Student t-test, **P = 0.0014 (untreated empty vector vs. treated empty vector), *P = 0.0100 (untreated PYK2 shRNA1 vs. treated PYK2 shRNA1), *P = 0.0240 (untreated PYK2 shRNA2 vs. treated PYK2 shRNA2), **P = 0.0013 (Empty vector vs. PYK2 shRNA1), ***P = 0.0007 (Empty vector vs. PYK2 shRNA2). N = 3 (3 replicates). **d** Graph representing percentage of CD44^High^/CD24^Low^ cells quantified by flow cytometry from SkBr3 control and PYK2 knockdowns cells metformin treated and untreated. Anova, *P = 0.0273 (Empty vector vs. PYK2 shRNA1), *P = 0.0182 (Empty vector vs. PYK2 shRNA2), **P = 0.0045 (Treated empty vector vs. treated PYK2 shRNA1), *P = 0.0211 (Treated empty vector vs. treated PYK2 shRNA2), **P = 0.0059 (untreated empty vector vs. treated empty vector), *P = 0.0208 (untreated PYK2 shRNA1*vs*. treated PYK2 shRNA1), **P = 0.0044 (untreated PYK2 shRNA2*vs*. treated PYK2 shRNA2). N = 3 (3 replicates). **e** Graph representing percentage of ALDH^High^ cells quantified by flow cytometry from SkBr3 control and PYK2 knockdowns cells metformin treated and untreated. Anova, *P = 0.0448 (Empty vector *vs*. PYK2 shRNA1), ***P = 0.0002 (Treated empty vector *vs*. treated PYK2 shRNA1), **P = 0.0033 (Treated empty vector *vs*. treated PYK2 shRNA1). N = 3 (3 replicates). **f** Cell invasion assay using SkBr3 control and PYK2 knockdowns cells metformin treated and untreated. and the corresponding data quantifying of number of invading cells (48 h with or without treatment). Anova ****P = < 0.0001. N = 3 (2 replicates). **g** Multivariate cox regression analysis of *PYK2* and *CD44* expression and their association with HER2+/ER−/PR- breast cancer patients’ survival over a 5-year term

Cancer stem-like cells are associated with chemoresistance and recurrence in patients following chemotherapy [35]. PYK2 has been shown to promote breast cancer stem cell enrichment in response to chemotherapy [36]. To investigate this possibility, sphere formation assays (self-renewal) were performed using untreated and treated control (pLKO.1 empty vector) and *PYK2* knockdown SkBr3 cells, and sphere size and number were assessed. The sphere size of PYK2 knockdown cells was significantly larger than the control, however, their number was significantly lower compared to the control (Fig. 6a, b and c). These differences were more significant in response to metformin treatment. Breast cancer stem cells are characterised by the phenotype CD44^High^/CD24^Low/−^ and ALDH^High^ [37] and therefore, we investigated if our sphere forming cells possess this phenotype, and if this correlates with the results of sphere formation assays. Indeed, our spheres were found to be enriched with cells that had a CD44^High^/CD24^Low/−^ and ALDH^High^ phenotype (Fig. 6d and e) Moreover, knockdown of *PYK2* resulted in a decrease of the percentage of CD44^High^/CD24^Low/−^ and ALDH^High^ cells, while metformin treatment promoted the enrichment of these cells (Fig. 6d and e). We also investigated the invasive capacity of untreated and metformin-treated control and *PYK2* knockdown SkBr3 spheres and we found that *PYK2* knockdown significantly reduced their invasive potential (Fig. 6f). The results above from initiation, self-renewal and invasion also indicate that the effect of metformin is PYK2-dependent. Finally, multivariate cox regression analysis demonstrates a significant reduction in cancer-specific survival over a 5-year term and that was associated with co-expression of *PYK2* and the stem cell marker *CD44* (Fig. 6g). This reduction was not affected by metformin treatment. Taken together, these results indicate that metformin promotes invasion mediated by HER2+/ER−/PR- breast cancer stem cells.

## Discussion

Although significant progress has been made in developing novel chemical compounds for cancer therapy, drug resistance is becoming a serious therapeutic challenge that hinders the efficiency of chemotherapies [1, 2]. As a result, drug resistance is responsible for cases of relapse and recurrence that lead to decreased survival in treated cancer patients. Thus, a better understanding of the mechanisms of chemoresistance will significantly contribute to the design and selection of more efficient compounds. Metformin is a small molecule drug that is successfully used for the treatment of diabetes type 2 and that has been suggested as a new therapeutic drug for several types of cancer. The choice of this drug for cancer therapy is based on epidemiologic reports that indicated that cancer patients affected with diabetes type 2 and treated with metformin, have reduced cancer risk and improved clinical outcomes [38–41]. Metformin exerts its anti-proliferative and pro-apoptotic functions by inhibiting mitochondrial complex I and by activating AMPK (5′ AMP-activated protein kinase). These actions lead to (i) the increase in endogenous levels of reactive oxygen species (ROS) and oxidative stress, resulting in cell death of cancer cells; (ii) activation of AMPK that inhibit mTOR pathway and activation of the tumour suppressor p53 [42–47].

Breast cancer is a heterogenous disease that can be classified into different molecular subtypes based on clinicopathology assessment, testing for hormonal (ER/PR) receptors and amplification of human epidermal growth factor receptor 2 (HER2). Treatment of breast cancer varies corresponding to the molecular subtype and involves endocrine therapies that target the oestrogen-receptor positive subtype, HER2 antibody-based targeting of HER2-positive subtype; and chemotherapy for the triple negative breast cancer [48–50]. Unfortunately, resistance to these therapies is also observed in aggressive breast cancer and needs for new chemotherapeutic compounds are required. In this regard, metformin has been suggested as potential drug for the treatment of breast cancer due to reduced cancer incidence in metformin-treated patients, and its anti-proliferative and pro-apoptotic effects on cancer cells.

In this study, we investigated the effect of metformin on cancer cell lines reflecting different breast cancer subtypes. Although, metformin effects were significantly noticeable in all studied breast cancer cell lines, we found that HER2+/ER−/PR- cancer cells were the most resistant to metformin. Interestingly, this resistance was associated with the activation of PYK2, a well-known molecule involved in proliferation, survival, migration and invasion. PYK2 has been involved in mediating downstream signalling of Integrins, GrRH, CCKR, inflammation-mediated by chemokine and cytokine pathways, via ERK/MAP kinase, PI3K/STAT3, WNT/b-catenin signalling pathways [26]. PYK2 expression has also been shown to increase in early and advanced ductal breast cancer, and this correlated with increased expression of HER2 [28]. The increase of PYK2 expression following metformin treatment may be explained by metformin activation of AMPK signalling that trigger the ERK/MAP kinase pathway leading to PYK2 activation [51, 52]. These observations prompted us to investigate further its role in HER2+/ER−/PR- cancer cell proliferation and invasion, and in response to metformin. We found that although metformin prevented proliferation, it also induced a PYK2-mediated cell invasion of HER2+/ER−/PR- cancer cells, and this was further confirmed by proteomic analysis. For instance, several proteins that are commonly downregulated in untreated and treated PYK2 knockdown cells such as the Rho GDP-dissociation inhibitor 2 GDIR2 (also known as ARHGDIB) are involved in regulating cell adhesion, migration and invasion. GDIR2 negatively regulates actin reorganisation, that is mediated by Rho family members, by regulating the GDP/GTP exchange reaction of Rho proteins [53, 54]. Another example is the inositol-3-phosphate synthase INO1, that is found downregulated, plays a role in p53-mediated growth suppression associated with myo-inositol biosynthesis pathway [55, 56]. Commonly upregulated proteins in untreated and treated PYK2 knockdown cells are associated with the upregulation of several proteins involved in cellular metabolism that is essential tumour growth and progression. Furthermore, we also demonstrate that a high expression of PYK2 is associated with a significant reduction in cancer-specific survival of HER2+/ER−/PR- breast cancer patients. PYK2 may also promote the emergence of HER2+/ER−/PR- cancer stem-like cells that may play a role in cancer chemoresistance, relapse and recurrence in patients who may receive metformin treatment in the future. This was further confirmed by demonstrating a correlation between reduced survival in pure HER2 breast cancer patients and expression of *PYK2* and the stem cell marker *CD44*. Although clinical trials are ongoing, testing this possibility in the future using samples from metformin-treated breast cancer patients and when available, will certainly shed light on potential induction of cancer invasion by metformin in treated patients.

## Conclusions

Our data indicates that metformin promotes HER2+/ER−/PR- breast cancer invasion through mechanisms involving PYK2, and that future treatments should consider potential complications resulting from metformin-based therapies.

## Additional files

Additional file 1: **Figure S1.** (A) Effect of metformin (25 mM) on apoptosis of SkBr3, MDA-MB-453 and MDA-MB-468. Anova, ***P = 0.0001. (B) (C) Immunoblot images representing PYK2 expression in SkBr3 and MDA-MB-453 control (pLKO.1 empty vector) and *PYK2* knockdown cells following cell culture passages week 2, 5 and 8. (D) Immunoblot images representing PYK2 expression in metformin treated and untreated MDA-MB-468, MDA-MB-231, BT-474 and MCF7 cell lines. (JPG 1083 kb)

Additional file 2: **Figure S2.** The difference in invasion and migration between metformin treated and untreated shRNA control and PYK-2 knockdown (KD) SkBr3 (A)(B) and MDA-MB-453 (C)(D) cells were calculated as ratios in comparison to the control of each cell line. Significance of the effect is calculated using two-way ANOVA in GraphPad prism with Tukey multiple comparison. Anova (SkBr3 proliferation), **P = 0.0059, *P = 0.0173, **P = 0.0078, *P = 0.0109; Anova (SkBr3 invasion), ****P = < 0.0001; Anova (MDA-MB-453 proliferation) ***P = 0.0003, ****P = < 0.0001, ***P = 0.0006, ***P = 0.0002; Anova (MDA-MB-453 invasion) ****P = < 0.0001. (JPG 1251 kb)

Additional file 3: **Figure S3.** (A) Heatmap representing the top 25 upregulated and downregulated proteins in untreated control and *PYK2* knockdown MDA-MB-453 cells. (B) Heatmap representing the top 25 upregulated and downregulated proteins in untreated control and *PYK2* knockdown metformin-treated MDA-MB-453 cells. (JPG 1535 kb)

Additional file 4: **Data S1.** Statistical values for Fig. 1a, b, c and d. (XLSX 41 kb)

Additional file 5: **Data S2.** Tables representing top 25 upregulated proteins and top 25 downregulated proteins in SkBr3 untreated control vs. untreated and treated *PYK2* knockdown samples with Fc 2 ≥ − 2, and confidence of 70%. (DOCX 26 kb)

Additional file 6: **Data S3.** Tables representing top 25 upregulated proteins and top 25 downregulated proteins in MDA-MB-453 untreated control vs. untreated and treated *PYK2* knockdown samples with Fc 2 ≥ − 2, and confidence of 70%. (DOCX 15 kb)

## Abbreviations

ALDH: Aldehyde Dehydrogenase
CD24: Cluster of Differentiation-24
CD44: Cluster of Differentiation-44
ER: Estrogen Receptor
HER2: Human Epidermal growth factor Receptor 2
PR: Progesterone Receptor
PYK2/PTK2B: Proline-Rich Tyrosine Kinase 2/Protein Tyrosine Kinase 2 Beta
