# Supplementary figures and images for: PYK2 promotes HER2-positive breast cancer invasion
Source: J Exp Clin Cancer Res. 2019 May 22;38:210. doi: 10.1186/s13046-019-1221-0 (PMC6532260; doi:10.1186/s13046-019-1221-0)

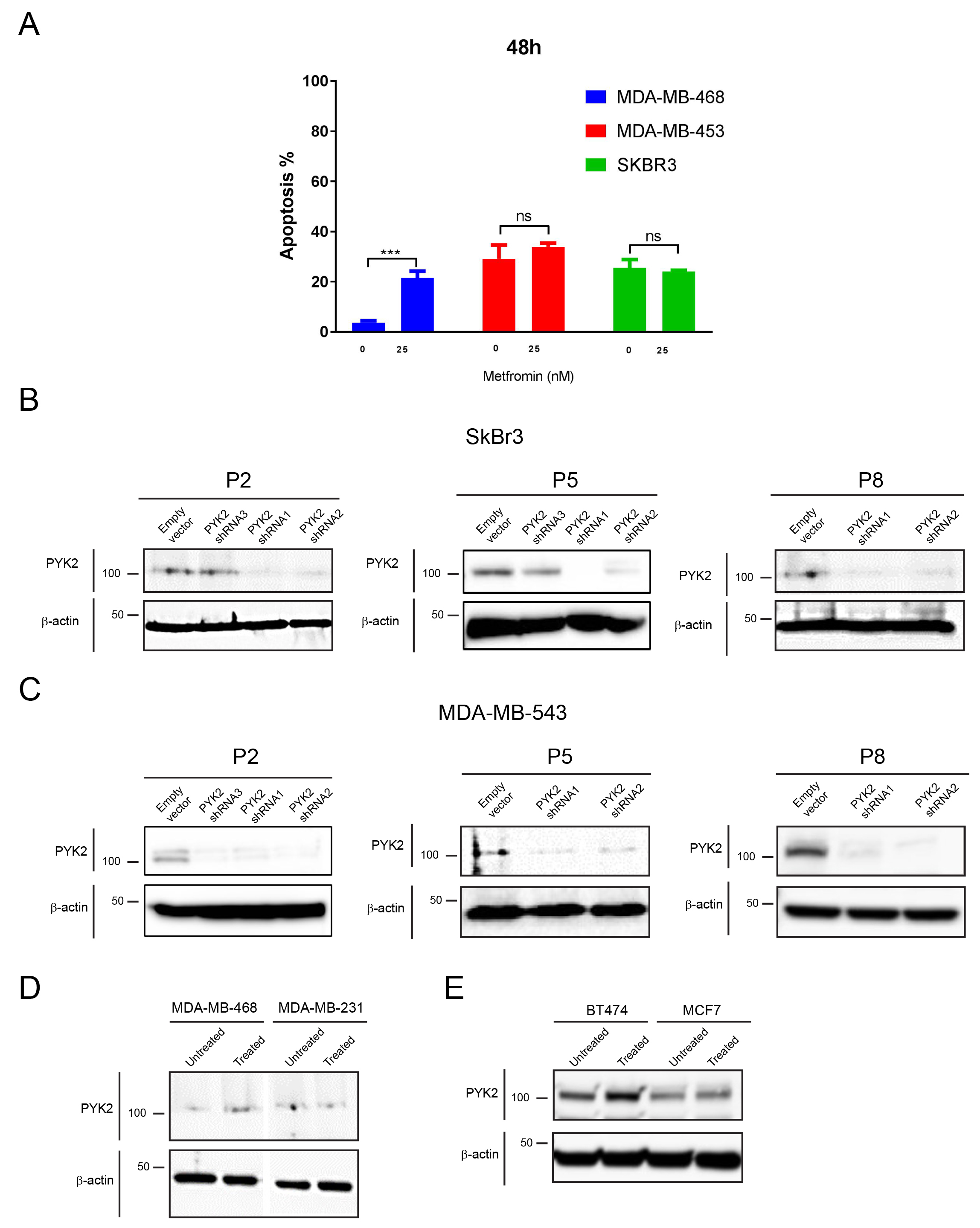

Supplement: Supplementary file 1 — Figure S1. (A) Effect of metformin (25 mM) on apoptosis of SkBr3, MDA-MB-453 and MDA-MB-468. Anova, ***P = 0.0001. (B) (C) Immunoblot images representing PYK2 expression in SkBr3 and MDA-MB-453 control (pLKO.1 empty vector) and PYK2 knockdown cells following cell culture passages week 2, 5 and 8. (D) Immunoblot images representing PYK2 expression in metformin treated and untreated MDA-MB-468, MDA-MB-231, BT-474 and MCF7 cell lines. (JPG 1083 kb) [file 13046_2019_1221_MOESM1_ESM.jpg]

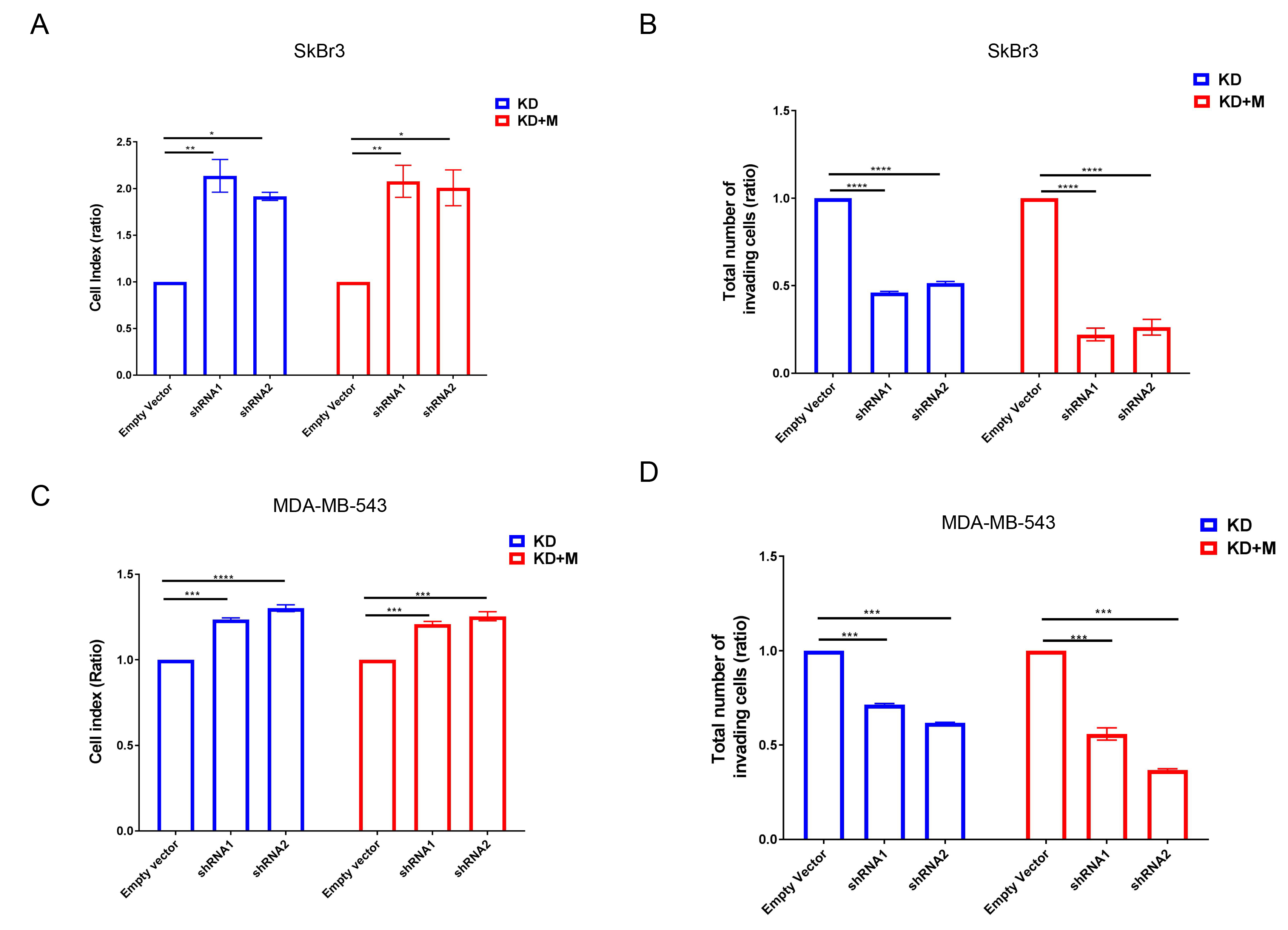

Supplement: Supplementary file 2 — Figure S2. The difference in invasion and migration between metformin treated and untreated shRNA control and PYK-2 knockdown (KD) SkBr3 (A)(B) and MDA-MB-453 (C)(D) cells were calculated as ratios in comparison to the control of each cell line. Significance of the effect is calculated using two-way ANOVA in GraphPad prism with Tukey multiple comparison. Anova (SkBr3 proliferation), **P = 0.0059, *P = 0.0173, **P = 0.0078, *P = 0.0109; Anova (SkBr3 invasion), ****P = < 0.0001; Anova (MDA-MB-453 proliferation) ***P = 0.0003, ****P = < 0.0001, ***P = 0.0006, ***P = 0.0002; Anova (MDA-MB-453 invasion) ****P = < 0.0001. (JPG 1251 kb) [file 13046_2019_1221_MOESM2_ESM.jpg]

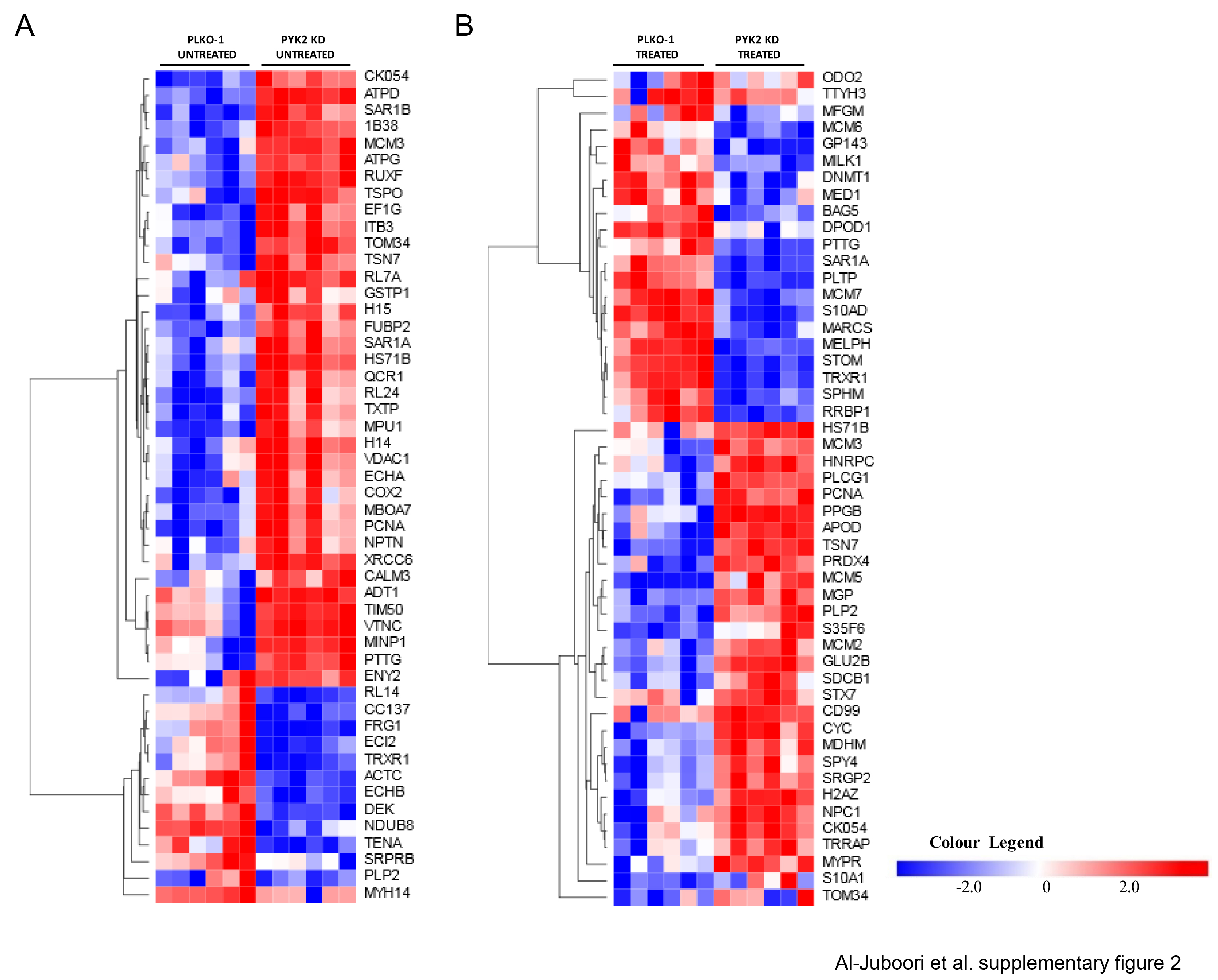

Supplement: Supplementary file 3 — Figure S3. (A) Heatmap representing the top 25 upregulated and downregulated proteins in untreated control and PYK2 knockdown MDA-MB-453 cells. (B) Heatmap representing the top 25 upregulated and downregulated proteins in untreated control and PYK2 knockdown metformin-treated MDA-MB-453 cells. (JPG 1535 kb) [file 13046_2019_1221_MOESM3_ESM.jpg]
