# Supplementary material for: PYK2 promotes HER2-positive breast cancer invasion
Source: J Exp Clin Cancer Res. 2019 May 22;38:210. doi: 10.1186/s13046-019-1221-0 (PMC6532260; doi:10.1186/s13046-019-1221-0)
Supplement: Supplementary file 5 — Data S2. Tables representing top 25 upregulated proteins and top 25 downregulated proteins in SkBr3 untreated control vs. untreated and treated PYK2 knockdown samples with Fc 2 ≥ − 2, and confidence of 70%. (DOCX 26 kb) [file 13046_2019_1221_MOESM5_ESM.docx]

Al-Juboori et al. supplementary data 1

| Protein Symbol | Description | Fold change | Confidence level |
| --- | --- | --- | --- |
| TOM5 | Mitochondrial import receptor subunit TOM5 homolog | 18.349 | 0.762 |
| SRSF4 | Serine/arginine-rich splicing factor 4 | 14.853 | 0.758 |
| H2AZ | Histone H2A.Z | 12.249 | 0.895 |
| AGR2 | Anterior gradient protein 2 homolog | 11.426 | 0.7908 |
| CPSM | Carbamoyl-phosphate synthase [ammonia], mitochondrial | 10.967 | 0.772 |
| GBG10 | Guanine nucleotide-binding protein G(I)/G(S)/G(O) subunit gamma-10 | 10.598 | 0.715 |
| LAGE3 | EKC/KEOPS complex subunit LAGE3 | 9.374 | 0.823 |
| MGST1 | Microsomal glutathione S-transferase 1 | 8.617 | 0.804 |
| NFYC | Nuclear transcription factor Y subunit gamma | 8.213 | 0.785 |
| PPOX | Protoporphyrinogen oxidase | 7.198 | 0.948 |
| HYEP | Epoxide hydrolase 1 | 7.111 | 0.824 |
| CALM3 | Calmodulin-3 | 7.052 | 0.718 |
| LYPD3 | Ly6/PLAUR domain-containing protein 3 | 6.878 | 0.789 |
| SAM50 | Sorting and assembly machinery component 50 homolog | 6.659 | 0.760 |
| MPC2 | Mitochondrial pyruvate carrier 2 | 6.587 | 0.789 |
| RABP1 | Cellular retinoic acid-binding protein 1 | 6.252 | 0.795 |
| CALX | Calnexin | 5.822 | 0.787 |
| QCR9 | Cytochrome b-c1 complex subunit 9 | 5.763 | 0.719 |
| MPCP | Phosphate carrier protein, mitochondrial | 5.503 | 0.710 |
| DHC24 | Delta (24)-sterol reductase | 5.376 | 0.772 |
| PCAT1 | Lysophosphatidylcholine acyltransferase 1 | 5.083 | 0.816 |
| VTM2L | V-set and transmembrane domain-containing protein 2-like protein | 4.847 | 0.789 |
| DHRS2 | Dehydrogenase/reductase SDR family member 2, mitochondrial | 4.753 | 0.876 |
| MGST3 | Microsomal glutathione S-transferase 3 | 4.579 | 0.776 |
| ZNT7 | Zinc transporter 7 | 4.540 | 0.875 |

| Protein Symbol | Description | Fold change | Confidence level |
| --- | --- | --- | --- |
| PEBP1 | Phosphatidylethanolamine-binding protein 1 | -2.890 | 0.902 |
| RSSA | 40S ribosomal protein SA | -2.946 | 0.885 |
| HSPB1 | Heat shock protein beta-1 | -2.962 | 0.844 |
| HEXA | Beta-hexosaminidase subunit alpha | -3.057 | 0.821 |
| RMD1 | Regulator of microtubule dynamics protein 1 | -3.133 | 0.822 |
| GDIR2 | Rho GDP-dissociation inhibitor 2 | -3.153 | 0.765 |
| TES | Testing | -3.195 | 0.843 |
| CNDP2 | Cytosolic non-specific dipeptidase | -3.228 | 0.753 |
| RL17 | 60S ribosomal protein L17 | -3.229 | 0.710 |
| S10A9 | Protein S100-A9 | -3.563 | 0.741 |
| CBX5 | Chromobox protein homolog 5 | -3.832 | 0.755 |
| CATB | Cathepsin B | -3.832 | 0.806 |
| INO1 | Inositol-3-phosphate synthase 1 | -3.844 | 0.739 |
| GPDA | Glycerol-3-phosphate dehydrogenase [NAD (+)], cytoplasmic | -4.107 | 0.761 |
| S10A4 | Protein S100-A4 | -4.239 | 0.721 |
| GPNMB | Transmembrane glycoprotein NMB | -4.831 | 0.724 |
| LXN | Latexin | -5.566 | 0.727 |
| TRFL | Lactotransferrin | -5.939 | 0.806 |
| FETUA | Alpha-2-HS-glycoprotein | -8.989 | 0.703 |
| RL35 | 60S ribosomal protein L35 | -9.105 | 0.831 |
| PSB8 | Proteasome subunit beta type-8 | -10.789 | 0.747 |
| LG3BP | Galectin-3-binding protein | -16.712 | 0.906 |
| CAPG | Macrophage-capping protein | -17.133 | 0.883 |
| ALBU | Serum albumin | -17.665 | 0.843 |
| F16P1 | Fructose-1,6-bisphosphatase 1 | -64.565 | 0.816 |

**Table 1. Top 25 upregulated proteins and 25 downregulated proteins in HER2 (SkBr3) cell line samples comparing untreated PLKO-1 and *PYK2* KD samples with Fc > +/- 2, and confidence > 70%/0.7 (SCIEX Oneomics confidence value).**

| Protein Symbol | Description | Fold change | Confidence level |
| --- | --- | --- | --- |
| SRSF4 | Serine/arginine-rich splicing factor 4 | 17.913 | 0.749 |
| GBG10 | Guanine nucleotide-binding protein G(I)/G(S)/G(O) subunit gamma-10 | 12.517 | 0.747 |
| CDS2 | Phosphatidate cytidylyltransferase 2 | 12.435 | 0.736 |
| TOM5 | Mitochondrial import receptor subunit TOM5 homolog | 11.294 | 0.779 |
| CPSM | Carbamoyl-phosphate synthase [ammonia], mitochondrial | 10.862 | 0.796 |
| H2AZ | Histone H2A.Z | 10.102 | 0.920 |
| AGR2 | Anterior gradient protein 2 homolog | 9.677 | 0.779 |
| PPOX | Protoporphyrinogen oxidase | 8.439 | 0.971 |
| HYEP | Epoxide hydrolase 1 | 8.375 | 0.825 |
| MGST1 | Microsomal glutathione S-transferase 1 | 8.102 | 0.807 |
| QCR9 | Cytochrome b-c1 complex subunit 9 | 7.351 | 0.749 |
| SAM50 | Sorting and assembly machinery component 50 homolog | 7.333 | 0.824 |
| ALG3 | Dol-P-Man: Man(5)GlcNAc(2)-PP-Dol alpha-1,3-mannosyltransferase | 6.978 | 0.782 |
| ZNT7 | Zinc transporter 7 | 6.974 | 0.789 |
| MPC2 | Mitochondrial pyruvate carrier 2 | 6.660 | 0.800 |
| CALM3 | Calmodulin-3 | 6.641 | 0.729 |
| RABP1 | Cellular retinoic acid-binding protein 1 | 6.610 | 0.808 |
| RCN3 | Reticulocalbin-3 | 6.375 | 0.777 |
| LYPD3 | Ly6/PLAUR domain-containing protein 3 | 6.334 | 0.721 |
| CALX | Calnexin | 6.136 | 0.873 |
| PCAT1 | Lysophosphatidylcholine acyltransferase 1 | 5.903 | 0.841 |
| SAR1A | GTP-binding protein SAR1a | 5.464 | 0.856 |
| DHC24 | Delta (24)-sterol reductase | 5.434 | 0.800 |
| SAR1B | GTP-binding protein SAR1b | 5.395 | 0.727 |
| DHRS2 | Dehydrogenase/reductase SDR family member 2, mitochondrial | 5.341 | 0.883 |

| Protein Symbol | Description | Fold change | Confidence level |
| --- | --- | --- | --- |
| RL7A | 60S ribosomal protein L7a | -3.210 | 0.802 |
| CNDP2 | Cytosolic non-specific dipeptidase | -3.230 | 0.755 |
| CATB | Cathepsin B | -3.267 | 0.781 |
| TES | Testing | -3.283 | 0.852 |
| RL24 | 60S ribosomal protein L24 | -3.681 | 0.905 |
| RL14 | 60S ribosomal protein L14 | -3.709 | 0.942 |
| GPDA | Glycerol-3-phosphate dehydrogenase [NAD (+)], cytoplasmic | -3.829 | 0.740 |
| TBB6 | Tubulin beta-6 chain | -3.835 | 0.822 |
| GDIR2 | Rho GDP-dissociation inhibitor 2 | -3.881 | 0.770 |
| INO1 | Inositol-3-phosphate synthase 1 | -4.017 | 0.741 |
| K2C7 | Keratin, type II cytoskeletal 7 | -4.060 | 0.710 |
| CBX5 | Chromobox protein homolog 5 | -4.163 | 0.831 |
| TRFL | Lactotransferrin | -5.657 | 0.701 |
| LXN | Latexin | -7.613 | 0.797 |
| S10A4 | Protein S100-A4 | -8.067 | 0.727 |
| INVO | Involucrin | -10.096 | 0.722 |
| RL35 | 60S ribosomal protein L35 | -10.733 | 0.820 |
| ZFAN1 | AN1-type zinc finger protein 1 | -11.261 | 0.7195 |
| EF1G | Elongation factor 1-gamma | -13.597 | 0.752 |
| PAGE5 | P antigen family member 5 | -14.763 | 0.713 |
| LG3BP | Galectin-3-binding protein | -16.528 | 0.900 |
| ALBU | Serum albumin | -16.538 | 0.831 |
| BTF3 | Transcription factor BTF3 | -18.724 | 0.730 |
| CAPG | Macrophage-capping protein | -20.770 | 0.837 |
| F16P1 | Fructose-1,6-bisphosphatase 1 | -26.012 | 0.834 |

**Table 2. Top 25 upregulated proteins and 25 downregulated proteins in HER2 (SkBr3) cell line samples comparing untreated PLKO-1 and Metformin-treated PYK2 KD samples with Fc > +/- 2, and confidence > 70%/0.7 (SCIEX Oneomics confidence value).**
