# Supplementary material for: PYK2 promotes HER2-positive breast cancer invasion
Source: J Exp Clin Cancer Res. 2019 May 22;38:210. doi: 10.1186/s13046-019-1221-0 (PMC6532260; doi:10.1186/s13046-019-1221-0)
Supplement: Supplementary file 6 — Data S3. Tables representing top 25 upregulated proteins and top 25 downregulated proteins in MDA-MB-453 untreated control vs. untreated and treated PYK2 knockdown samples with Fc 2 ≥ − 2, and confidence of 70%. (DOCX 15 kb) [file 13046_2019_1221_MOESM6_ESM.docx]

| **Protein Symbol** | **Description** | **Fold change** | **Confidence level** |
| --- | --- | --- | --- |
| **RUXF** | Small nuclear ribonucleoprotein F | -1.646 | 0.735 |
| **XRCC6** | X-ray repair cross-complementing protein 6 | -1.652 | 0.736 |
| **ACTC** | Actin, alpha cardiac muscle 1 | -1.663 | 0.802 |
| **EF1G** | Elongation factor 1-gamma | -1.669 | 0.781 |
| **GSTP1** | Glutathione S-transferase P | -1.726 | 0.770 |
| **FUBP2** | Far upstream element-binding protein 2 | -1.798 | 0.895 |
| **FRG1** | Protein FRG1 | -1.817 | 0.716 |
| **MINP1** | Multiple inositol polyphosphate phosphatase 1 | -1.854 | 0.803 |
| **DEK** | Protein DEK | -1.858 | 0.807 |
| **PCNA** | Proliferating cell nuclear antigen | -1.962 | 0.796 |
| **TOM34** | Mitochondrial import receptor subunit TOM34 | -1.987 | 0.716 |
| **CK054** | Ester hydrolase C11orf54 | -2.084 | 0.854 |
| **ENY2** | Transcription and mRNA export factor ENY2 | -2.111 | 0.894 |
| **RL7A** | 60S ribosomal protein L7a | -2.189 | 0.787 |
| **MCM3** | DNA replication licensing factor MCM3 | -2.197 | 0.776 |
| **RL24** | 60S ribosomal protein L24 | -2.291 | 0.878 |
| **TRXR1** | Thioredoxin reductase 1, cytoplasmic | -2.615 | 0.816 |
| **CC137** | Coiled-coil domain-containing protein 137 | -2.743 | 0.729 |
| **RL14** | 60S ribosomal protein L14 | -2.841 | 0.882 |
| **TENA** | Tenascin | -3.582 | 0.856 |
| **HS71B** | Heat shock 70 kDa protein 1B | -3.673 | 0.878 |
| **H15** | Histone H1.5 | -3.895 | 0.767 |
| **PLP2** | Proteolipid protein 2 | -4.246 | 1.000 |
| **H14** | Histone H1.4 | -4.274 | 0.759 |
| **VTNC** | Vitronectin | -5.846 | 0.839 |

**Table 1:** Top 50 upregulated and downregulated proteins in HER2 (MDA-MB-453) cell line samples comparing untreated PLKO-1 and *PYK2* KD samples with Fc 2 ≥ -2, and confidence of 70%.

| **Protein Symbol** | **Description** | **Fold change** | **Confidence level** |
| --- | --- | --- | --- |
| **ADT1** | ADP/ATP translocase 1 | 13.499 | 0.742 |
| **SAR1A** | GTP-binding protein SAR1a | 10.812 | 0.778 |
| **NDUAC** | NADH dehydrogenase [ubiquinone] 1 alpha subcomplex subunit 12 | 10.489 | 0.734 |
| **RHOG** | Rho-related GTP-binding protein RhoG | 9.006 | 0.706 |
| **T106B** | Transmembrane protein 106B | 8.776 | 0.713 |
| **SAR1B** | GTP-binding protein SAR1b | 8.292 | 0.846 |
| **VDAC1** | Voltage-dependent anion-selective channel protein 1 | 8.020 | 0.735 |
| **1B38** | HLA class I histocompatibility antigen, B-38 alpha chain | 7.796 | 0.810 |
| **COX2** | Cytochrome c oxidase subunit 2 | 7.624 | 0.733 |
| **IFRD1** | Interferon-related developmental regulator 1 | 7.482 | 0.800 |
| **ITB3** | Integrin beta-3 | 7.329 | 0.776 |
| **PTTG** | Pituitary tumor-transforming gene 1 protein-interacting protein | 6.870 | 0.761 |
| **QCR1** | Cytochrome b-c1 complex subunit 1, mitochondrial | 6.767 | 0.727 |
| **VKOR1** | Vitamin K epoxide reductase complex subunit 1 | 6.217 | 0.868 |
| **ECHA** | Trifunctional enzyme subunit alpha, mitochondrial | 6.134 | 0.778 |
| **NDUS8** | NADH dehydrogenase [ubiquinone] iron-sulfur protein 8, mitochondrial | 6.110 | 0.789 |
| **COX20** | Cytochrome c oxidase protein 20 homolog | 5.844 | 0.841 |
| **PHB** | Prohibitin | 5.651 | 0.904 |
| **NB5R3** | NADH-cytochrome b5 reductase 3 | 5.631 | 0.751 |
| **TSN7** | Tetraspanin-7 | 5.508 | 0.721 |
| **ECI2** | Enoyl-CoA delta isomerase 2, mitochondrial | 5.405 | 0.757 |
| **PHB2** | Prohibitin-2 | 5.390 | 0.919 |
| **5NTD** | 5'-nucleotidase | 5.192 | 0.785 |
| **CYB5B** | Cytochrome b5 type B | 5.099 | 0.713 |
| **T4S1** | Transmembrane 4 L6 family member 1 | 5.072 | 0.743 |

**Table2:** Top 50 upregulated and downregulated proteins in HER2 (MDA-MB-453) cell line samples comparing untreated PLKO-1 and Metformin-treated *PYK2* KD samples with Fc 2 ≥ -2, and confidence of 70%.
